# NKX2-2 based nuclei sorting on frozen human archival pancreas enables the enrichment of islet endocrine populations for single-nucleus RNA sequencing

**DOI:** 10.1186/s12864-024-10335-w

**Published:** 2024-04-30

**Authors:** Gengqiang Xie, Maria Pilar Toledo, Xue Hu, Hyo Jeong Yong, Pamela Sandoval Sanchez, Chengyang Liu, Ali Naji, Jerome Irianto, Yue J. Wang

**Affiliations:** 1 Department of Biomedical Sciences, College of Medicine, Florida State University, 1115 West Call Street, Tallahassee, FL 32306 USA; 2 Department of Surgery, Hospital of the University of Pennsylvania, Philadelphia, PA USA

**Keywords:** Single-nucleus RNA-seq, NKX2-2, Fluorescence-activated nuclei sorting (FANS), Frozen archival human pancreas, Islets, Endocrine population enrichment

## Abstract

**Background:**

Current approaches to profile the single-cell transcriptomics of human pancreatic endocrine cells almost exclusively rely on freshly isolated islets. However, human islets are limited in availability. Furthermore, the extensive processing steps during islet isolation and subsequent single cell dissolution might alter gene expressions. In this work, we report the development of a single-nucleus RNA sequencing (snRNA-seq) approach with targeted islet cell enrichment for endocrine-population focused transcriptomic profiling using frozen archival pancreatic tissues without islet isolation.

**Results:**

We cross-compared five nuclei isolation protocols and selected the citric acid method as the best strategy to isolate nuclei with high RNA integrity and low cytoplasmic contamination from frozen archival human pancreata. We innovated fluorescence-activated nuclei sorting based on the positive signal of NKX2-2 antibody to enrich nuclei of the endocrine population from the entire nuclei pool of the pancreas. Our sample preparation procedure generated high-quality single-nucleus gene-expression libraries while preserving the endocrine population diversity. In comparison with single-cell RNA sequencing (scRNA-seq) library generated with live cells from freshly isolated human islets, the snRNA-seq library displayed comparable endocrine cellular composition and cell type signature gene expression. However, between these two types of libraries, differential enrichments of transcripts belonging to different functional classes could be observed.

**Conclusions:**

Our work fills a technological gap and helps to unleash frozen archival pancreatic tissues for molecular profiling targeting the endocrine population. This study opens doors to retrospective mappings of endocrine cell dynamics in pancreatic tissues of complex histopathology. We expect that our protocol is applicable to enrich nuclei for transcriptomics studies from various populations in different types of frozen archival tissues.

**Supplementary Information:**

The online version contains supplementary material available at 10.1186/s12864-024-10335-w.

## Background

The pancreas is a complex organ with two major elements: the exocrine component and the endocrine component. The exocrine component, which is 95% of the pancreatic mass, produces and secretes digestive enzymes into the small intestine. The endocrine component of the pancreas, which is less than 5% of the pancreatic mass, secretes hormones into the blood to regulate glucose homeostasis. The functional and structural unit of the endocrine pancreas is the islet of Langerhans. Islets contain five endocrine cell types: glucagon-producing alpha cells, insulin-producing beta cells, somatostatin-producing delta cells, ghrelin-producing epsilon cells, and pancreatic polypeptide-producing PP cells. The endocrine pancreas is the central focus of research in type 1 (T1D) and type 2 diabetes (T2D) [1–3].

Because of the diverse cellular components of the endocrine pancreas, bulk assays cannot dissect the cell type-specific biological signatures. In recent years, there has been an explosion of single-cell RNA-seq (scRNA-seq) studies aiming to profile in depth the human endocrine pancreas in development and disease at the single-cell resolution [4–13]. These studies have brought unprecedented insights into islet biology [13]. However, the starting material of these scRNA-seq studies is mostly freshly isolated pancreatic islets — a limited and expensive resource that comes with several challenges: (1) The long and laborious islet isolation procedure may alter the islet-cell cellular states and their gene expression profile [14]. (2) Post isolation, islets are transported to recipient laboratories and cultured in vitro for days before enzymatic dissociation for scRNA-seq experiments [15]. These procedures themselves may induce cellular stress that results in changes in gene expression programs [16, 17]. (3) Successful islet isolation relies on optimal collagenase digestion [18]. Various factors influence the efficiency of collagenase digestion, notably the integrity and composition of the peri-insular basement membrane, which consists of different types of collagens and other extracellular matrix proteins [19]. The variations in the peri-insular basement membrane in younger donors, in donors with T1D, and in donor pancreata with various pathologies render islet isolation extremely challenging [20, 21]. (4) The complete dependence on islets isolated from fresh pancreatic tissues for scRNA-seq misses the opportunity to utilize the rich frozen archival tissues available in pancreatic tissue biobanks such as the Network for Pancreatic Organ Donors with Diabetes (nPOD) [22].

Recently, a few protocols were developed for scRNA-seq profiling on fixed cells [23, 24] or frozen islets [25, 26]. However, these protocols still rely on the isolation of live islet cells in the first place for targeted interrogation of islet endocrine cells, and hence do not overcome the limitations of islet isolation detailed above. To effectively utilize the existing large collections of biobank pancreatic tissues with no islet isolation, we present our workflow of single-nucleus RNA-seq (snRNA-seq) combining optimized nuclei isolation with fluorescence-activated nuclei sorting (FANS) based on NKX2-2 to enrich pancreatic endocrine cells from frozen human pancreata. Our method bypasses the need for isolating islets and makes it possible to utilize frozen archived pancreatic tissues including tissues from various pancreatic pathologies for transcriptomic profiling focused on the endocrine system.

## Methods

### Nuclei isolation from frozen human pancreas

Five protocols potentially compatible with nuclei isolation and RNA sequencing were cross-compared: Frankenstein protocol [27], ATAC-seq protocol [28], sNucDrop-seq protocol [29], GRO-seq protocol [30, 31], and citric acid protocol [32]. Frozen mouse pancreata were used for protocol comparison. Each protocol was performed as described in their original publications. In all protocols, the nuclei isolation steps were performed on ice. Detailed protocols are included in the Supplementary method and step-by-step protocol.

After isolating nuclei using each protocol, a portion of the sample was counted with a hemocytometer to assess nuclei yield. To evaluate nuclei purity, the remaining nuclei were labeled with DAPI (1 μg/ml) and celltracker red (1:2000, ThermoFisher Scientific, C34552) for 30 min on ice and imaged under an Olympus microscope at 40 × magnification.

Based on the yield, purity, and mRNA quality of isolated nuclei (see RESULTS), we selected the citric acid method for all subsequent experiments using frozen archival human pancreas as input.

### Nuclei labeling and purification

Isolated nuclei were immediately fixed and permeabilized by ice cold methanol at -20 °C for 10 min. Nuclei were washed twice in the resuspension buffer (1× PBS, 1% BSA + 10% glycerol + 0.2 U/μl RNase inhibitor). Nuclei were then labeled with the primary antibody against NKX2-2 (DSHB, 74.5A5, 1:100) and Cy3 donkey-anti-mouse secondary antibody (Jackson ImmunoResearch, 715-165-151, 1:200). Subsequently, nuclei were stained with DAPI at a final concentration of 1 μg/ml. Right before FANS, nuclei were filtered through a 35 μm cell strainer. BD FACSAria with a 100 μm nozzle was used for targeted nuclei sorting. Sorted nuclei were loaded onto a 10× Genomics Chromium Next GEM Chip G (10X Genomics, PN1000127). Detailed procedure is included in the step-by-step protocol.

### snRNA-seq library preparation and sequencing

snRNA-seq libraries were prepared from three donors. Details of the donors’ demographic information is shown in Table S1. Libraries were prepared following the manufacturer’s protocol (Chromium Next GEM Single Cell 3’ Reagent Kits v 3.1, CG000315). Libraries were sequenced on an Illumina Novaseq 6000 instrument.

### snRNA-seq and scRNA-seq data analysis

FASTQ files were aligned to GRCh38-3.0.0 using Cell Ranger V.5.0.1 and Include introns = TRUE. The raw gene expression matrices were input to Soup X 1.6.2 [33] for ambient RNA removal using *autoEstCont* to automatically estimate the contamination fraction. Contamination was removed from the original count matrix to generate a corrected gene expression matrix. All the downstream analysis was performed in Seurat V4.3.0 [34], with the corrected gene expression matrix as input. Data quality control (QC), integration, dimension reduction, clustering, and cell type calling were performed with Seurat similarly as previously described [35, 36].

For the snRNA-seq data, the individual dataset was first filtered with minimal reads of 200 and a maximum percentage of mitochondrial reads of 5%. To compare the snRNA-seq data from the frozen pancreas between non-enriched and NKX2-2+ enriched populations, the two libraries were normalized with *SCTransform* and integrated using the anchor-based method by sequentially calling for *SelectIntegrationFeatures*, *PrepSCTIntegration*, *FindIntegrationAnchors*, and *IntegrateData*, all with default parameters. *RunPCA*, *RunUMAP*, *FindNeighbors*, and *FindClusters* were then performed on the integrated assay. A resolution of 0.4 was used for cell clustering. Cell type classification was based on the expression of canonical pancreatic markers in each cluster: GCG for alpha cells, INS for beta cells, SST for delta cells, GHRL for epsilon cells, PPY for PP cells, CFTR for ductal cells, CPA2 for acinar cells, SPARC for fibroblasts, VWF for endothelial cells, PTPRC for immune cells, and BRCA1 for proliferating cells. One population coexpressing multiple cell type markers was categorized as doublets.

Raw scRNA-seq (HPAP080_sc) FASTQ data was downloaded from PANC-DB [37]. The scRNA-seq data were aligned to GRCh38-3.0.0 and corrected for ambient RNA as described above for snRNA-seq data. The resulting gene expression matrix was filtered with minimal reads of 200 and a maximum percentage of mitochondrial reads of 15%. The scRNA-seq (HPAP080_sc) and snRNA-seq (HPAP080_sn) data were then integrated and annotated using the same process described above for integrating snRNA-seq datasets. For visualization, the two datasets were projected to the Human Pancreas Reference from Azimuth using *FindTransferAnchors* and *MapQuery* with default parameters.

The proportions of spliced and unspliced counts in the snRNA-seq and scRNA-seq libraries were computed by invoking *velocyto run10x* [38] with cellranger prebuilt GRCh38-3.0.0 GTF file and default parameters.

### Differential expression analysis

Differential expression analyses were performed using the limma-trend method [39] and a threshold of FDR < 0.01 and log2FC > 1 was used in all comparisons to select significantly differentially expressed genes.

To derive signature genes in pancreatic endocrine cells, the panc8 dataset available as Seurat Data [6–8, 11, 40] was utilized. The design matrix was coded as model.matrix(~ Condition + Tech). Condition includes endocrine cells (alpha, beta, delta, epsilon, and PP) and others (ductal, acinar, endothelial, fibroblast, macrophage, mast, and schwann); and Tech indicates the different single-cell chemistries. A contrast fit was applied to compare endocrine cells to others. To prioritize nuclear-enriched proteins, the resulting list was intersected with the list of transcription factors downloaded from the Human Protein Atlas [41].

To calculate marker gene cell-type specificity, we computed the *tau* score based on the average gene expression of each marker gene in each cell type. The *tau* score was calculated as follows [42, 43]:

$$\tau =\frac{\sum_{i=1}^{n}\left(1-\widehat{{x}_{i}}\right)}{n-1};\widehat{{x}_{i}}=\frac{{x}_{i}}{\underset{1\le i\le n}{{\text{max}}}\left({x}_{i}\right)}.$$

Where Xi is the average expression of the gene in cell type i and n is the number of cell types. Cell types here refer to the cell type in each donor condition (for example, beta cells in T2D and beta cells in T1D are considered two different cell types). A *tau* score of 0 means ubiquitous expression whereas a *tau* score close to 1 means the transcript is highly cell-type specific.

Cell type signatures were derived in the snRNA-seq and scRNA-seq data separately. Within each dataset, the design matrix was coded as model.matrix(~ 0 + Celltype) with Celltype being different cell type labels. To compare an endocrine cell type of interest, e.g., alpha cells, to all of the other endocrine cell types, the contrast matrix was coded as makeContrasts(alpha_vs_others = Celltypealpha - (Celltypebeta + Celltypedelta + Celltypepp)/3, levels = colnames(design)). Integrated expressions of the union of the cell type signature genes were used to construct the heatmap in Fig. 4F.

To compare snRNA-seq with scRNA-seq data, differential expression analysis was performed in each cell type with a design matrix as model.matrix(~ 0 + tech) with tech being snRNA-seq or scRNA-seq.

### Rank-rank hypergeometric overlap

Rank–rank hypergeometric overlap (RRHO) is a threshold-free method aiming to compare gene expression profiles across two gene lists that were ranked by the degree of differential expression from two separate differential expression analyses [44]. The original RRHO method was further modified to improve the interpretability when the differential expression patterns are discordant in the two gene lists [45]. The input gene lists for RRHO were ranked by the log2FC (computed by limma-trend, see above) comparing the gene expressions of each endocrine cell type with all the other endocrine cell types in the snRNA-seq or scRNA-seq library.

### Immunostaining

Frozen pancreatic sections were fixed with ice-cold methanol for 10 min at -20°C. The following primary antibodies and dilutions were used: anti-INSULIN (Invitrogen, 701265, 1:300), anti-GLUCAGON (Santa Cruz Biotechnology, sc-514592-AF546, 1:100), anti-SOMATOSTATIN (Santa Cruz Biotechnology, sc-7819, 1:300), anti-GHRELIN (Santa Cruz Biotechnology, sc-10368, 1:500), anti-PANCREATIC POLYPEPTIDE (Abcam, ab77192, 1:500), anti-NKX2-2 (DSHB, 74.5A5, 1:25). The following secondary antibodies were used: Cy2-anti-rabbit (Jackson ImmunoResearch, 711–225-152), Cy2-anti-goat (Jackson ImmunoResearch, 705–225-147), Cy3-anti-mouse (Jackson ImmunoResearch, 715–165-151), Cy5-anti-rabbit (Jackson ImmunoResearch, 711–175-152), and Cy5-anti-mouse (Jackson ImmunoResearch, 115–175-207). All secondary antibodies were applied at 1:200 dilution. Slide scanning images were taken with an Olympus microscope at 20x/0.75NA. Confocal images were captured with a Zeiss at 20x/0.75NA.

## Results

### Comparison of five nuclei isolation methods

**Fig. 1:**
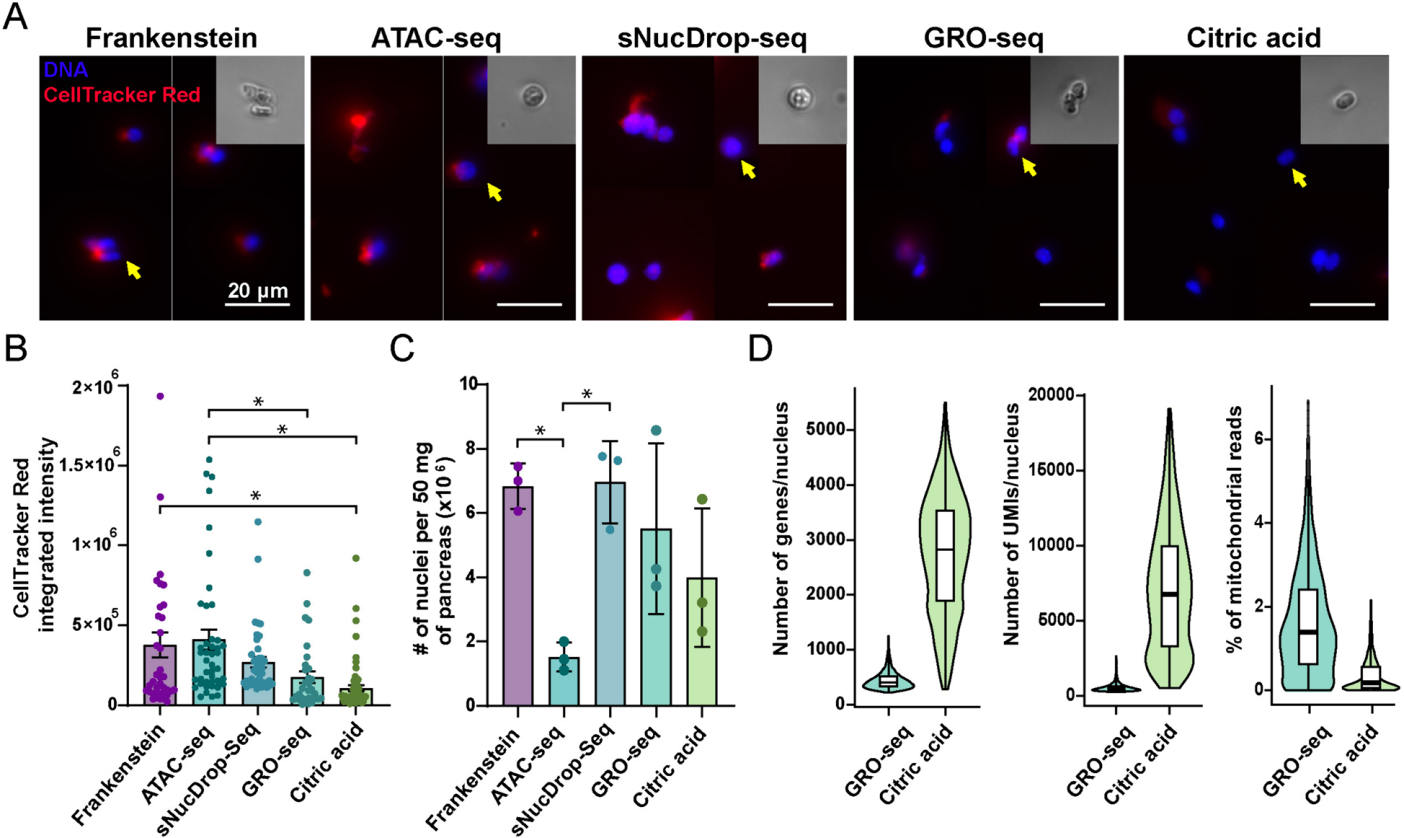
Citric acid method is the best method to isolate nuclei from frozen pancreata. **A** Cross-comparison of five different nuclei isolation protocols. Isolated nuclei were labeled with DAPI (DNA, blue) and CellTracker Red (red) and imaged under a 20× epifluorescent microscope. Scale bars correspond to 20 μm. Inserts show zoomed-in bright field images of the nuclei pointed by arrows. **B** Quantification of the CellTracker Red signals in nuclei isolated with different methods. * indicates adjusted *P* value < 0.05 with one way ANOVA and Tukey post hoc. GRO-seq and citric acid methods generate intact and high purity nuclei with the lowest cytoplasmic contaminations. **C** Nuclei yield from each isolation protocol, normalized to 50 mg of pancreatic tissue. Error bars indicate standard errors. * indicates adjusted *P* value < 0.05 with one way ANOVA and Tukey post hoc. **D** Violin plots showing distributions of the number of genes/nucleus, number of UMIs/nucleus, and percentage of mitochondrial reads in the snRNA-seq libraries with nuclei isolated with GRO-seq method or citric acid method. Box plots inside the violins display the distribution of the first quartile, median, and third quartile, as well as minimum and maximum

Several groups have demonstrated the feasibility of isolating nuclei from frozen tissues followed by RNA-seq [32, 46–50]. However, existing nuclei isolation protocols vary in yield, purity, and procedure complexity, relying on homemade solutions or commercial kits. To explore the best strategy to isolate nuclei from frozen archival pancreatic tissues, we compared five widely used nuclei isolation methods: (1) the Frankenstein method [27]; (2) the ATAC-seq method [28, 51]; (3) the sNucDrop-seq protocol method [29]; (4) the GRO-seq method [30, 31]; and (5) the citric acid method [32, 52]. To evaluate the performance of the different protocols, we utilized snap-frozen mouse pancreata. To assess whether the isolated nuclei were free of cytoplasmic contamination, we stained them with DAPI to label DNA and CellTracker Red to label cytoplasm. We observed that all methods preserved nuclei integrity, as shown by the clear nuclear boundaries and minor blebbing under the bright field (Fig. 1A). However, the Frankenstein, ATAC-seq, and sNucDrop-seq methods produced nuclei with higher cytoplasmic contaminations compared with the GRO-seq and citric acid protocols (Fig. 1A and B). Furthermore, the ATAC-seq method had lower nuclei yield (Fig. 1C).

We proceeded to prepare two snRNA-seq libraries with nuclei isolated from frozen archival human pancreata using the two methods that generated the cleanest nuclei — the GRO-seq and the citric acid method. The complexity and purity of the snRNA library prepared with the citric acid method were significantly higher compared with the library of the GRO-seq method. This is supported by the higher number of genes, higher number of unique molecular identifiers (UMIs), and the lower percentage of mitochondrial reads per nucleus in the citric acid library compared with the GRO-seq library (Fig. 1D). Furthermore, the ambient RNA percentage estimate was 25% for the GRO-seq library and 3% for the citric acid library based on SoupX [33], indicating compromised nuclei quality in the former and good quality in the latter. We subsequently used the citric acid method in all the following experiments.

### NKX2-2 as a pan-endocrine marker in the human pancreas

**Fig. 2:**
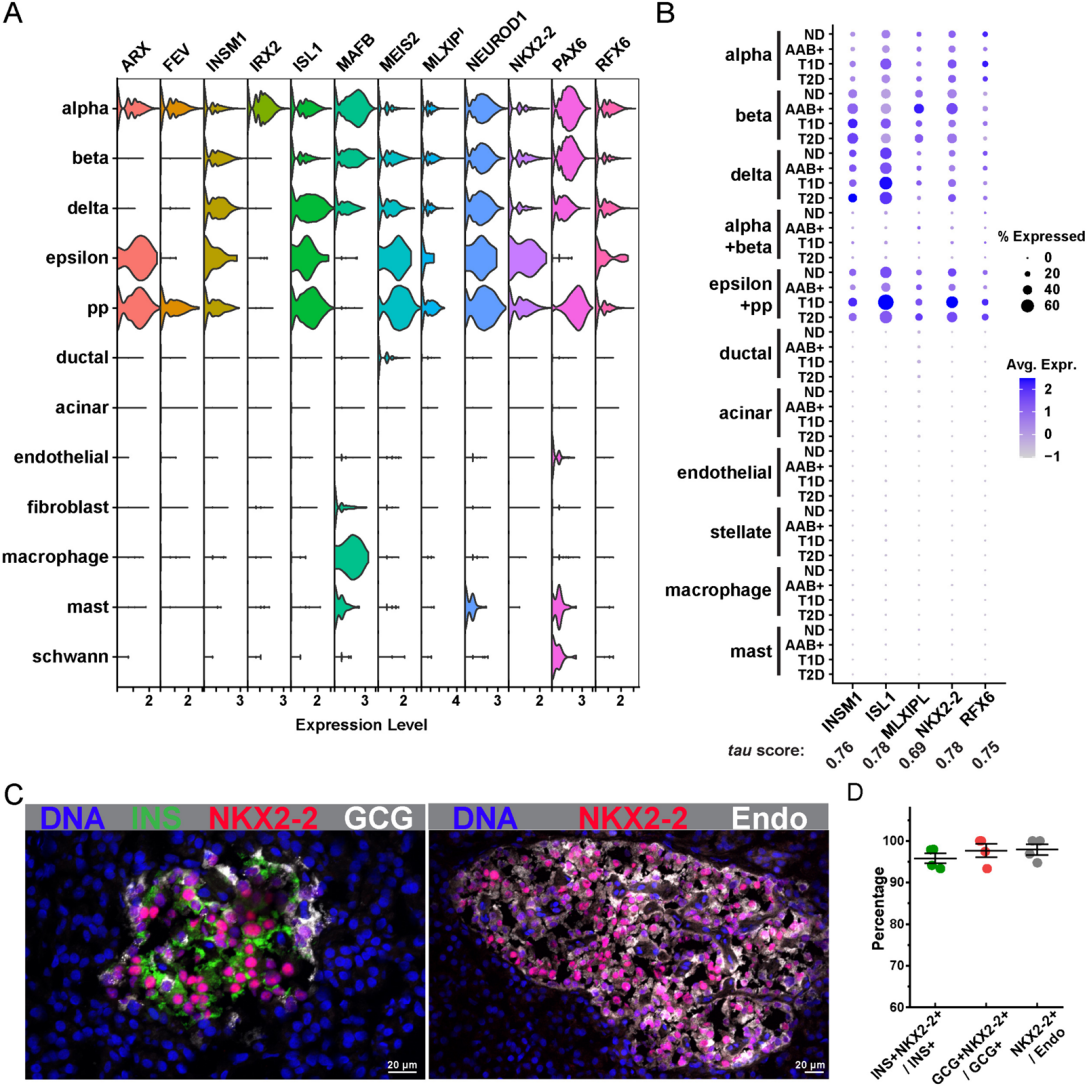
NKX2-2 as a pan-endocrine marker in the human pancreas across normal and different pathological conditions. (**A**) The RNA expression levels of top 12 endocrine-cell enriched transcription factors in different human pancreatic cell types. INSM1, ISL1, MLXIPL, NKX2-2, and RFX6 show exclusive and ubiquitous expressions in the endocrine cells. **B** Dot plot summarizes the expression of candidate endocrine markers in the pancreatic cells from controls (ND), autoantibody-positive (AAB+), T1D, and T2D donors. The size of the dot represents the percentage of cells expressing the marker genes, while the color of the dot indicates the average expression of the marker genes across all cells. Tau score for each marker is shown under the gene name. **C** Immunofluorescent labeling in the human pancreatic tissue confirms NKX2-2 as a pan-endocrine marker. Nuclei are labeled with DAPI (DNA, blue). Left, tissue is co-labeled with INSULIN (INS, green), NKX2-2 (red), and GLUCAGON (GCG, white). Right, tissue is co-labeled with NKX2-2 (red) and pan-endocrine cocktail (Endo, white) with a mixture of anti-INSULIN, GLUCAGON, SOMATOSTATIN, GHRELIN, and PANCREATIC POLYPEPTIDE antibodies. Scale bars correspond to 20 μm. **D** Quantification of the co-expression of NKX2-2 and endocrine markers. Each dot represents one individual islet

Islet cells constitute only 5% of cells of the pancreas. To efficiently capture nuclei of islet cells from the total pancreatic nuclei pool, we reasoned that a pan-endocrine nuclear marker could be used to enrich the nuclei of the target population. To identify such a marker, we explored the panc8 data, which contains merged scRNA-seq data derived from eight human pancreatic datasets [6–8, 11, 40]. We performed differential expression analysis comparing the gene expression differences between the endocrine cells and all the other pancreatic cell types and intersected the resulting differential expression gene list with the list of transcription factors to prioritize markers expressed in the nuclei. Twelve markers emerged from this analysis: ARX, FEV, INSM1, IRX2, ISL1, MAFB, MEIS2, MLXIPL, NEUROD1, NKX2-2, PAX6, and RFX6. Among them, INSM1, ISL1, MLXIPL, NKX2-2, and RFX6 display a pan-endocrine expression pattern (Fig. 2A). To ensure the broad usability of the pancreatic endocrine markers, we investigated their expressions in pancreatic cells associated with different pathologies including autoantibody-positive (AAB+), T1D, and T2D. We utilized scRNA-seq data generated with 65 donors from HPAP that were recently annotated by the Gaulton group [37, 53]. We confirmed that NKX2-2 is consistently expressed in islet endocrine cells in control, AAB+ , T1D, or T2D pancreata, with one of the highest gene specificity *tau* scores (Fig. 2B). To further validate the ubiquitous expression of NKX2-2 at the protein level in the islet endocrine cells, we performed immunostaining with an anti-NKX2-2 antibody on frozen human pancreatic tissue sections (Fig. 2C). The NKX2-2+ signal was detected in the nuclei of close to all islet endocrine cells, as shown in Fig. 2D. Our expression analysis of NKX2-2 in the human pancreas agrees with what was previously reported in mice [54] and nominates NKX2-2 as a pan-endocrine marker in the pancreas in normal and pathological conditions across species.

### Fluorescence-activated nuclei sorting (FANS) to enrich pancreatic endocrine population for targeted snRNA-seq profiling

**Fig. 3:**
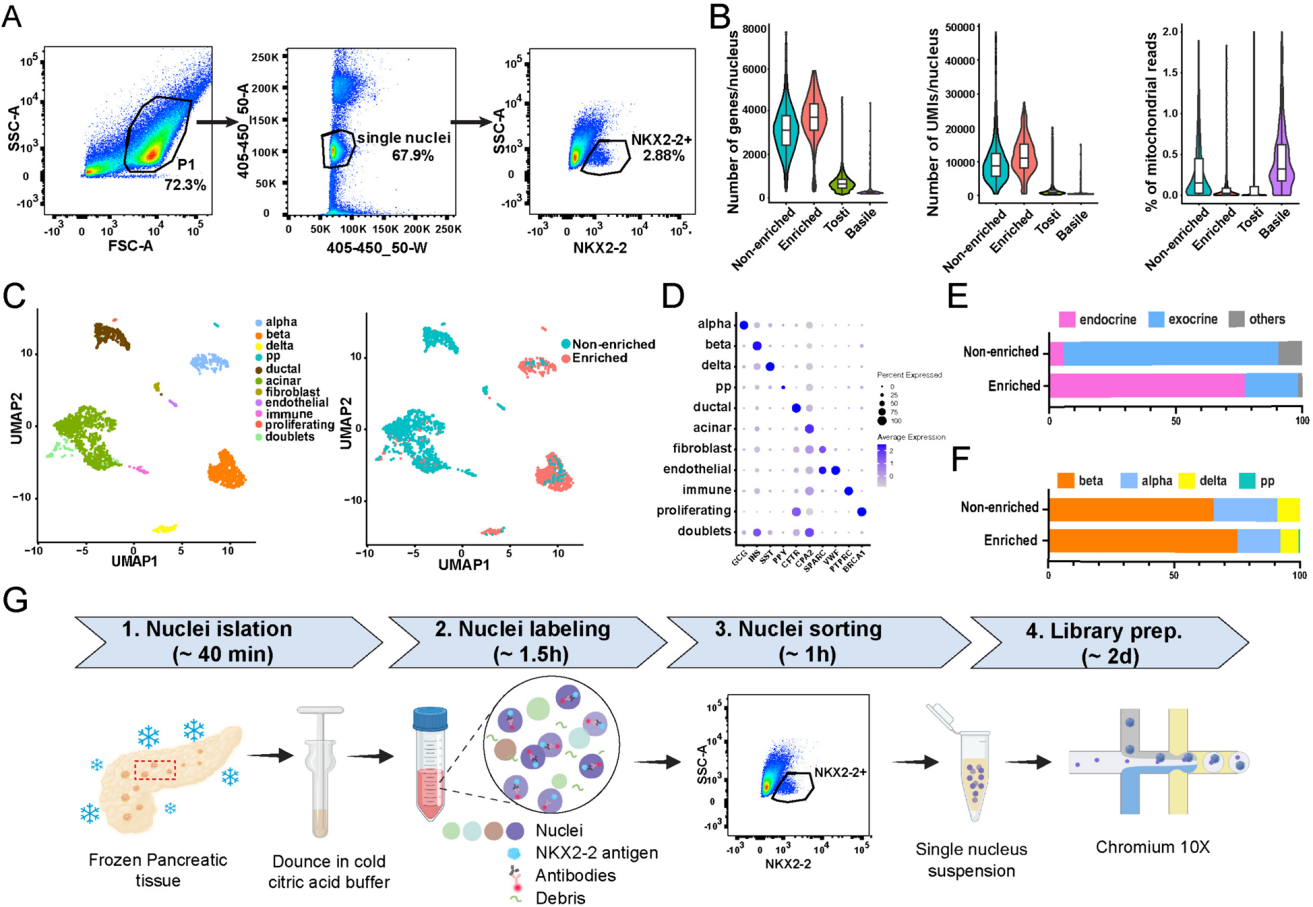
snRNA-seq with NKX2-2-based enrichment enables transcriptomic profiling of endocrine population from frozen archival human pancreata without islet isolation. **A** Sequential gating strategy to enrich nuclei from endocrine population by FANS. Nuclei are first gated in FSC and SSC (P1) to exclude debris and aggregates. Nuclei in P1 are then gated based on DAPI signal area versus width to select single nuclei. Endocrine nuclei are then selected and sorted based on the positive expression of NKX2-2. Population percentages from a representative experiment are shown next to each gate. **B** Composite violin and box plots showing distributions of the number of genes/nucleus, number of UMIs/nucleus, and percentage of mitochondrial reads in our snRNA-seq libraries with or without NKX2-2 based enrichment compared with Tosti et al. [32] and Basile et al. [25]. **C** UMAP embedding with cells colored according to cell type (left) and samples (right). **D** Dot plot illustrating the expression of marker genes in each cell type. **E** The proportions of endocrine and exocrine cells in the two snRNA-seq libraries. **F** Endocrine cell composition in the two snRNA-seq libraries. **G** The overall experimental workflow of snRNA-seq with islet-cell enrichment

To demonstrate the feasibility of using an anti-NKX2-2 antibody to enrich nuclei of pancreatic islets from frozen archival pancreatic tissue in the snRNA-seq experiment, we processed two snRNA-seq libraries using frozen pancreatic tissue from one donor. Nuclei were isolated with the citric acid method and methanol fixed and permeabilized immediately after isolation. Methanol was used because it not only permeabilizes the nuclei and allows antibodies to access nuclear epitopes but has also been shown to preserve the integrity of mRNA [55, 56]. For one sample (non-enriched), the nuclei were subjected to FANS to isolate intact single nuclei based solely on DAPI signals; for the other sample (enriched), nuclei were sorted to enrich NKX2-2+ endocrine population (Fig. 3A). Next, both samples were processed following the standard 10× Genomics scRNA-seq library preparation procedure. We evaluated the quality of these two snRNA-seq libraries and benchmarked these two libraries against two recently published snRNA-seq datasets [25, 32] (Fig. 3B). The complexity and purity of our snRNA libraries compared favorably with the current field standard, evidenced by the higher number of genes and UMIs detected and the lower percentage of mitochondrial reads per nucleus in our libraries compared with the other two published datasets (Fig. 3B). To be noted, the frozen pancreas we used here had a relatively long cold ischemia time (20 h, Table S1), representative of the general timeline of human tissue and organ harvesting and preservation.

We integrated the snRNA-seq data from the non-enriched and enriched samples and annotated cell types based on marker gene expression (Fig. 3C and D). All major pancreatic cell types could be detected from our dataset. Approximately 3% of the cells expressed multiple cell type markers and were annotated as doublets. We confirmed that targeted enrichment based on NKX2-2 labeling increased the proportion of endocrine cells from 5.3% to 76.7% (> 14-fold enrichment) (Fig. 3E). Comparing the fraction of each endocrine cell type in these two samples, we observed that with or without NKX2-2-based enrichment, the cellular compositions were highly similar between the two samples (correlation coefficient *r* = 0.98) (Fig. 3F), confirming that NKX2-2 antibody-based FANS is not biasing for or against a certain endocrine population. The Tosti, et al. [32] dataset contains cell type annotation from the original authors, enabling the comparison of cell type proportions (Figure S1). We observed that the proportions of endocrine/exocrine/others cells in the Tosti et al. dataset (average from 6 donors) were similar to our non-enriched sample, with endocrine cells constituting 5–6% of all cells. This result further confirmed the relatively low abundance of the endocrine cells in the ensemble pancreatic cellular space and underlined the importance of endocrine population enrichment for targeted molecular profiling. We conclude that our protocol offers increased flexibility to generate high-quality gene expression snRNA-seq libraries of islets from frozen archival pancreatic tissues. Figure 3G summarizes the sample processing workflow.

### Comparison of snRNA-seq and scRNA-seq modalities from the same donor

**Fig. 4:**
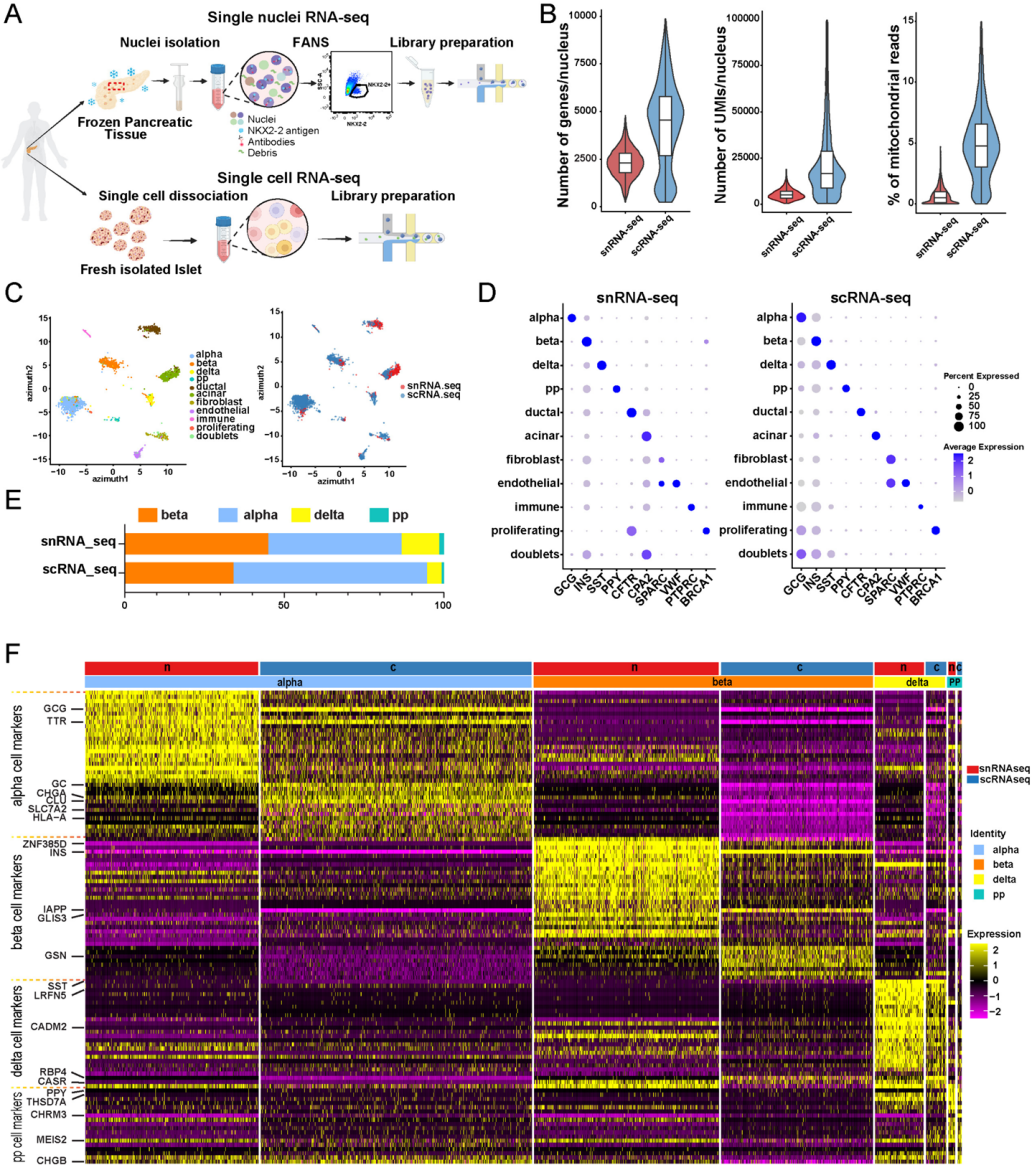
Comparison of snRNA-seq and scRNA-seq libraries from the same donor. **A** Experimental design for generating endocrine population enriched snRNA-seq library from frozen human pancreas and scRNA-seq library from freshly isolated islets, both from the same donor. **B** Composite violin and box plots showing distributions of the number of genes/nucleus, number of UMIs/nucleus, and percentage of mitochondrial reads in the two libraries. **C** UMAP embedding with cells colored according to cell type (left) and samples (right). **D** Dot plot illustrating the expression of marker genes in each cell type in the snRNA-seq (left) and scRNA-seq (right) libraries. **E** Endocrine cell composition in the two libraries. **F** Heatmap showing the relative expression of cell type markers in the two libraries. n, snRNA-seq library. c, scRNA-seq library. The rows of the heatmap correspond to genes and columns to cells. Canonical markers of each cell type are extracted from van Gurp et al. [57] and labeled next to the corresponding row

Having confirmed that our sample preparation procedure generates good quality data, we proceeded to prepare a pancreatic endocrine population enriched snRNA-seq library using frozen archival pancreas from one donor and compared it with the scRNA-seq library prepared using freshly isolated human islets from the same donor (Fig. 4A). We compared the snRNA-seq and scRNA-seq modalities from the same donor in order to control for gene expression variations originating from donor heterogeneity.

After removing low-quality nuclei/cells, we retained 5,938 nuclei in the snRNA-seq dataset and 2,645 cells in the scRNA-seq dataset. All cells passing QC were used in the downstream comparative analysis. As expected, scRNA-seq detected larger numbers of genes and UMI per cell and had a higher percentage of mitochondria reads compared with snRNA-seq (Fig. 4B). We aligned the two datasets for visualization by projecting these data to the Azimuth Human Pancreas Reference (Fig. 4C) [58]. Both datasets recovered all major pancreatic cell types as distinguished by specific marker gene expressions (Fig. 4D). Within the endocrine population, these two libraries showed strong similarities in cell type compositions (*r* = 0.89) (Fig. 4E). 0.5% of nuclei in the snRNA-seq library and 6.8% of the cells in the scRNA-seq library expressed multiple cell type markers and were annotated as doublets (Fig. 4D). We next extracted cell type specific signatures in each modality (Table S2). We observed that cell type markers display similar expressions between snRNA-seq and scRNA-seq libraries (Fig. 4F). We used RRHO2 [44, 45] to formally compare the expression of cell type signature genes in snRNA-seq and scRNA-seq datasets. We observed significant concordant patterns between the two libraries in genes upregulated or downregulated in each endocrine cell type compared with the rest of the endocrine cells (Figure S2).

**Fig. 5:**
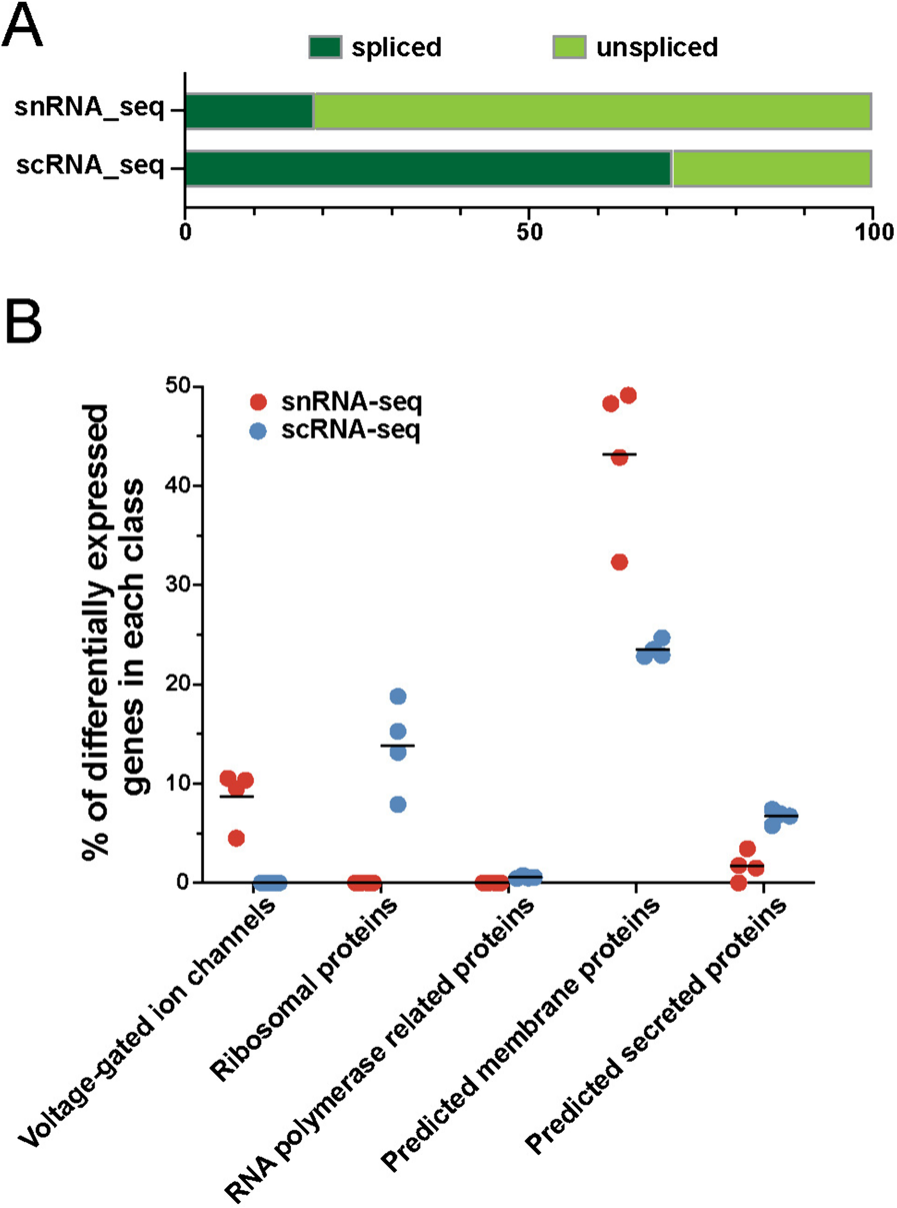
Differences in transcriptomics captured between the snRNA-seq and scRNA-seq modalities. **A** Percentage of spliced/unspliced reads in each library. **B** Differential enrichment of genes in different functional classes between the two types of libraries. Each dot represents one of the four endocrine cell types (alpha, beta, delta, PP) in the snRNA-seq or scRNA-seq data. Y axis corresponds to the percentage of genes significantly higher expressed in snRNA-seq (red) or scRNA-seq (blue) dataset that belongs to each functional class. Only significantly differentially enriched functional classes are shown

Next, we evaluated the differences between snRNA-seq and scRNA-seq in the transcripts they recovered. We observed that 81% of all transcripts detected in the snRNA-seq data were unspliced whereas 71% of transcripts in the scRNA-seq data were spliced (Fig. 5A). The differences in the percentage of spliced/unspliced transcripts in the two modalities are similar to what was reported in the brain tissues [59] and reflect the differences in transcripts’ subcellular origins. To further understand differential transcripts enriched in nuclear versus whole-cell transcriptomes, we performed differential expression analysis between these two modalities in each cell type (Table S3). We categorized the differentially expressed genes into 19 feature classes based on the human protein atlas annotation (https://www.proteinatlas.org/humanproteome/proteinclasses) [41]. Common to all endocrine cell types, genes higher expressed in snRNA-seq were significantly enriched (multiple t-test, FDR < 5%) in the classes of voltage-gated ion channels and membrane proteins; while genes higher expressed in the scRNA-seq data were significantly enriched (multiple t-test, FDR < 5%) in the categories of ribosomal proteins, RNA polymerase related proteins and secreted proteins (Fig. 5B). The differential enrichment of mRNAs between nuclei and whole cells is likely reflective of different transcripts’ transcription and processing rates, nuclear export speed, and half-lives.

## Discussion

Over the last decade, scRNA-seq has driven major advances in our understanding of human islet biology [13]. However, because of the reliance on freshly isolated islets, scRNA-seq studies have serious time constraints and limited source materials. In this work, we optimized a nuclei isolation method with targeted enrichment to characterize the transcriptional landscape of pancreatic endocrine cells from frozen human pancreatic tissues without islet isolation. The success of nuclei isolation is less influenced by pancreatic microenvironment changes including inflammation, breakdown of the islet basement membrane, or the fibrosis seen in various pancreatic diseases and in donors with advanced ages. Hence, single-nucleus transcriptomic workflow has built-in advantages to profile endocrine populations from samples that are difficult for islet isolation including those from young donors or donors with various pancreatic pathologies including T1D and T2D. Indeed, using two young donors in our study (Table S1), we demonstrate the feasibility of our workflow to obtain high-quality endocrine population enriched transcriptomics data from samples where islet isolations are challenging. Moreover, snRNA-seq is better for capturing intrinsic cellular states because it utilizes snap-frozen tissues, and the entire procedure is conducted on ice [17]. Our method presents an exciting opportunity for retrospective studies on the islet cells using frozen archival human tissues from biobanks.

Our protocol is easy to set up and does not use commercial kits or ultracentrifugation. The procedure can be separated into two major parts: (1) nuclei isolation from frozen tissues and (2) target population enrichment. The nuclei isolation step involves lysing cells at low pH in a hypotonic citric acid buffer. No RNase inhibitor is needed in the buffer because the acidic environment and the citric acid’s metal-chelating property effectively inhibit the activities of RNase [52, 60]. One major advantage of this protocol is that it does not require the genetic labeling of target populations, making it exceptionally adaptable for applications involving human tissues and various other organisms. Our protocol is versatile for target population enrichment and this enrichment is not limited to cell types. Any intranuclear epitopes that can be targeted by antibodies (single antibody or antibody combinations), including signaling molecules and metabolic proteins, can be used to enrich populations of interest. We expect the protocol to be easily adapted to isolate and enrich nuclei from a wide range of populations in the pancreas and in different organs/tissues. Moreover, our procedure is theoretically compatible with other methods for single-cell transcriptomic and proteomic profiling [24, 61], with the optional change of fixative from methanol to paraformaldehyde to increase cross-linking.

Our method benchmarks favorably against two recently published snRNA-seq datasets using frozen human pancreata [32] or frozen human islets [25] (Fig. 3B). The application of detergent-free citric acid buffer likely avoids uncontrollable lysing of cells when tissue conditions are less than ideal. Furthermore, the FANS step in our protocol not only enriches for target population but also enriches intact single nuclei [46, 62, 63], hence explaining the observed higher quality of our snRNA-seq libraries compared with Tosti et al. [32] despite the usage of the same citric acid method. To be noted, the pancreatic tissues used in the experiment had 13–20 h of cold ischemia time and between 1.3 to 15.3 years of storage time (Table S1). The fact that we were able to obtain good-quality snRNA-seq data from all these samples underscores the robustness of our experimental procedure.

Collectively using three donors, we demonstrate the feasibility of our workflow using NKX2-2 based nuclei sorting on frozen human archival pancreas to enrich islet endocrine populations for snRNA-seq. NKX2-2 is a highly conserved homeobox transcription factor [64]. In mice, Nkx2-2 is broadly expressed in the pancreatic progenitor cells during early embryogenesis and gradually restricted to Neurog3+ endocrine progenitor cells and later to mature islet endocrine cells [64, 65]. In humans, the expression of NKX2-2 is absent in early progenitor cells and appears in differentiated pancreatic endocrine cells of all types after 8 weeks post-conception [66, 67]. Here, we confirm that NKX2-2 is expressed in almost all human pancreatic endocrine cells (Fig. 2). Furthermore, the stability of NKX2-2 expression across control, AAB+ , T1D, and T2D conditions (Fig. 2B) makes it an excellent marker to enrich endocrine cells in various pancreas endocrine pathologies. In fact, in our laboratory, we have used the same workflow on frozen T1D pancreata and obtained high-quality snRNA-seq libraries on endocrine populations (data not shown).

The snRNA-seq data and scRNA-seq data in our study have comparable cellular compositions and cell type marker gene expressions (Figs. 4F, S1, Table S2). Nonetheless, differences exist in the transcripts captured by the two modalities (Figs. 4B and 5, Table S3). Compared to scRNA-seq, snRNA-seq recovers lower numbers of genes, has lower mitochondrial reads, and enriches unspliced RNA transcripts (Figs. 4B and 5A). Hence snRNA-seq provides a nuclear-centric transcriptional view complementary to the whole-cell perspective of scRNA-seq. Differences between these two modalities can be also observed at the individual gene level. In the scRNA-seq data, the canonical hormone markers for different endocrine cell types (INS, GCG, SST, PPY for alpha, beta, delta, and PP cells correspondingly) are the top enriched markers based on log2 fold change. In the snRNA-seq data, INS and SST remain as the top enriched markers in beta and delta cells respectively. However, in alpha cells, PTPRT ranked first while GCG ranked fifth. In PP cells, CHRM3 ranked first while PPY ranked fourth (Table S2). PTPRT and CHRM3 were also among the top enriched cell-type specific markers in a recent study using snRNA-seq from isolated human islets [26]. PTPRT and CHRM3 both encode membrane proteins, a protein class that is significantly enriched in the snRNA-seq dataset compared with scRNA-seq dataset (Fig. 5B). A study revealed that transcripts encoding membrane proteins have a long residing time in the nuclei, potentially explaining their relatively higher abundance in the nuclei compared with whole cells [68].

## Conclusions

In summary, we develop a FANS protocol on human frozen archival pancreatic tissues to enrich islet endocrine populations for single nucleus transcriptomic profiling. Our study opens doors to retrospective mappings of endocrine cell dynamics in frozen archival pancreatic tissues of complex histopathology.

### Supplementary Information

**Supplementary Material 1.**

**Supplementary Material 2.**

**Supplementary Material 3.**

**Supplementary Material 4.**

**Supplementary Material 5.**

**Supplementary Material 6.**

**Supplementary Material 7.**

## Data Availability

The datasets supporting the conclusions of this article are available be downloaded from GEO under the accession number GSE252614.
