# Supplementary material for: NKX2-2 based nuclei sorting on frozen human archival pancreas enables the enrichment of islet endocrine populations for single-nucleus RNA sequencing
Source: BMC Genomics. 2024 Apr 30;25:427. doi: 10.1186/s12864-024-10335-w (PMC11059690; doi:10.1186/s12864-024-10335-w)
Supplement: Supplementary file 1 — Supplementary Material 1. [file 12864_2024_10335_MOESM1_ESM.docx]

**Detailed nuclei isolation protocol**

- Frankenstein protocol

Approximately 50 mg of frozen pancreatic tissue was chopped into small pieces with a razor blade. Tissues were then dounced in a 2 ml glass douncer with 1 ml of Nuclei EZ lysis buffer (Millipore Sigma, NUC101-1KT) using pestle B for 10-20 strokes. Next, homogenized tissue was transferred into a 15 ml tube and incubated with an additional 2 ml of Nuclei EZ lysis buffer for 5 minutes on ice. The homogenate was then filtered with a 70 μm cell strainer. Nuclei were pelleted by centrifugation at 500g for 5 minutes at 4oC. Nuclei were incubated again with 1.5 ml of Nuclei EZ lysis buffer for 5 minutes on ice. Subsequently, nuclei were pelleted and washed twice in nuclei wash and resuspension buffer (1x PBS, 1% BSA, 0.2 U/μl RNase inhibitor). Finally, nuclei were filtered through a 35 μm cell strainer.

- ATAC-seq protocol

Approximately 50 mg of frozen pancreatic tissue was chopped into small pieces with a razor blade. Tissues were then dounced in a 7 ml glass douncer with 2 ml of homogenization buffer (5 mM CaCl2, 3 mM Mg(Ac)2, 10 mM Tris-HCl, pH 7.5, 0.028 mM PMSF, 0.29 mM β-mercaptoethanol, 320 mM sucrose, 0.1 mM EDTA, 0.1% Igepal) using pestle A for 10 strokes and pestle B for 20 strokes. Large chunks of tissues were pelleted at 100g for 1 minute at 4oC. The supernatant was transferred to a 15 ml conical tube. Samples were mixed with 1 volume (2 ml) of 50% iodixanol solution in homogenization buffer, and under layered with 3 ml of 29% iodixanol solution in homogenization buffer, followed by another underlayer with 3 ml of 35% iodixanol solution in homogenization buffer. Next, samples were spun for 20 minutes at 3,000g at 4oC. Finally, the nuclei band between the 35% iodixanol and the 29% iodixanol was collected and washed once with 1 ml of wash buffer (1x PBS, 0.2 U/μl RNase inhibitor).

- sNucDrop-seq protocol

For the sNucDrop-seq protocol, approximately 50 mg of frozen pancreatic tissue was chopped into small pieces with a razor blade. Tissues were then dounced in a 2 ml glass douncer with 1 ml of homogenization buffer (320 mM sucrose, 5 mM CaCl2, 10 mM Tris-HCl, pH 8.0, 3 mM Mg(Ac)2, 0.1% Triton, 0.1 mM EDTA, and 1 tablet of protease inhibitor per 50 ml of homogenization buffer) using pestle B for 15 strokes. Subsequently, nuclei were sequentially filtered by a 100 μm cell strainer and a 40 μm cell strainer. Nuclei were then pelleted at 700g for 10 minutes at 4oC. Finally, nuclei were resuspended in the resuspension buffer (1x PBS, 0.01% BSA) and filtered again by a 40 μm cell strainer.

For the GRO-seq protocol, approximately 50 mg of frozen pancreatic tissue was chopped into small pieces with a razor blade. Tissues were then dounced in a 7 ml glass douncer with 3 ml of swelling buffer (10 mM Tric-HCl, pH 7.5, 2 mM MgCl2, 3 mM CaCl2, 0.2 U/μl RNase inhibitor) using pestle A for 10 strokes. Next, tissues were incubated for 20 minutes in swelling buffer on ice and then dounced again with pestle B for 20 strokes. An additional 3 ml of swelling buffer was then added and nuclei were filtered through a 100 μm cell strainer. Nuclei were pelleted at 400g for 10 minutes at 4oC and resuspended in 2 ml of swelling buffer with 10% glycerol and 0.2 U/μl RNase inhibitor. 2 ml of lysis buffer (swelling buffer with 10% glycerol, 1% Igepal, 0.2 U/μl RNase inhibitor) was then added drop by drop. Nuclei were then incubated in lysis buffer on ice for 5 minutes. Finally, nuclei were washed twice in lysis buffer and pelleted at 900g for 5 minutes at 4oC.

- Citric acid protocol

Please refer to the step-by-step protocol.
