# Supplementary material for: NKX2-2 based nuclei sorting on frozen human archival pancreas enables the enrichment of islet endocrine populations for single-nucleus RNA sequencing
Source: BMC Genomics. 2024 Apr 30;25:427. doi: 10.1186/s12864-024-10335-w (PMC11059690; doi:10.1186/s12864-024-10335-w)
Supplement: Supplementary file 2 — Supplementary Material 2. [file 12864_2024_10335_MOESM2_ESM.docx]

**NKX2-2 based nuclei sorting on frozen human archival pancreas enables the enrichment of islet endocrine populations for single nucleus RNA sequencing**

**Step-by-step experimental protocol**

**Before starting**

* Pre-chill 7 ml douncers in a 4 °C refrigerator.

* Pre-chill razor blade on ice.

* Cool down a table-top centrifuge to 4 ^o^C.

* Prepare the following buffers and chill them on ice.

Buffer preparation:

1. Citric-acid buffer, keep on ice (buffer can be stored at 4 ^o^C for months).

Sucrose 0.25 M

Citric Acid 25 mM

2. Citric-acid density buffer, keep on ice (buffer can be stored at 4 ^o^C for months).

Sucrose 0.88 M

Citric Acid 25 mM

3. Resuspension buffer, keep on ice (buffer without RNaseIn* and DTT can be stored at 4 ^o^C for months).

KCl 25 mM

MgCl_2_ 3 mM

Tris-HCl (pH 7.5) 50 mM

RNase inhibitor (add fresh) 0.4 U/μl

DTT (add fresh) 1 mM

4. 100% methanol

5. Staining buffer (buffer without RNaseIn can be stored at 4 ^o^C for months).

PBS 1 x

BSA 1%

Glycerol 10%

RNase inhibitor (add fresh) 0.2 U/μl

6. Collection buffer (buffer without RNaseIn can be stored at 4 ^o^C for months).

PBS 1 x

BSA 1%

RNase inhibitor (add fresh) 0.2 U/μl

**Nuclei isolation**

1. Weigh the frozen pancreatic tissue before starting**. Cut tissue into pieces < 0.3 cm with a cold razor blade on dry ice.
2. Add 3 ml of *citric-acid buffer* and dounce once with the loose pestle (pestle A).
3. Incubate tissue on ice for 5 min.
4. Homogenize with 5-10 strokes of pestle A.
5. Incubate tissue on ice for 5 min.
6. Homogenize with 3-5 strokes of pestle A and 5 more strokes of pestle B.
7. Filter tissue through 100 μm cell strainer into a 15 ml conical tube.
8. Use 3 ml of citric acid buffer to wash the douncer and filter the solution through with 100 μm cell strainer into the same conical tube.
9. Centrifuge for 5 min at 500 g at 4 ^o^C.
10. Remove supernatant.
11. Resuspend nuclei pellet with 3 ml of *citric-acid buffer*.
12. Centrifuge for 5 min at 500 g at 4 ^o^C.
13. Remove supernatant.
14. Resuspend nuclei with 1 ml of *citric-acid buffer*.
15. Use a P1000, go to the bottom of the nuclei suspension, and gently add 1 volume (1 ml) of *citric-acid density buffer*. Do not resuspend.
16. Centrifuge at 4 ^o^C, 1000 g for 10 min.
17. Remove supernatant.

**Be very careful. The nuclei pellet is loose in high concentration sucrose solution. Better to leave some solution at the bottom!**

1. Resuspend in 1 ml of *citric-acid buffer*.
2. Centrifuge for 5 min at 500 g at 4 ^o^C.
3. Remove supernatant.
4. Reconstitute the nuclei in 1 ml of *resuspension buffer* and count the nuclei.

**Antibody labeling**

All steps are performed on ice.

1. Spin down the sample at 900 g for 5 min at 4^o^C.
2. Remove the supernatant but leave 100 μl of buffer behind. Resuspend nuclei with the residual buffer in the tube.
3. Add 1 ml of ice-cold *methanol* dropwise to prevent aggregation.
4. Fix and permeabilize nuclei in methanol at -20 °C for 10 min.
5. By the end of 10 min, spin down the nuclei at 900 g for 5 min at 4 °C.
6. Collect the supernatant into another tube.
7. Wash nuclei with 500 μl of *staining buffer*. Spin at 900 g for 5 min at 4 °C. Spin the supernatant collected in the previous step together.
8. Combine the nuclei pellet from the tubes by resuspending in a total of 1.2ml of *staining buffer*. Split the sample into 3 tubes with the following labels and volumes:

Tube 1: non-staining, 100 μl

Tube 2: DAPI only, 100 μl

Tube 3: DAPI + NKX2.2, 1 ml

1. Leave tube 1 and 2 on ice.
2. Spin tube 3 at 900 g for 5 min at 4 ^o^C.
3. Remove supernatant from tube 3.
4. Incubate nuclei from tube 3 with 300 μl of the NKX2-2 primary antibody (DSHB, 74.5A5, 1:100 dilution in *staining buffer*) on ice for 30 min.
5. Wash twice with 500 μl of *staining buffer* for tube 3, spin at 900 g for 5 min at 4°C.
6. Incubate tube 3 nuclei with 300 μl of secondary Cy3 donkey-anti-mouse antibody (Jackson ImmunoResearch, 715-165-151, 1: 200 dilution in *staining buffer*) plus DAPI (1 mg/ml stock) (1:1000 dilution) on ice for 30 min.
7. Simultaneously, incubate tube 2 with DAPI (1:1000 dilution) on ice for 30 min.
8. Wash tube 2 and tube 3 twice with 500 μl of *staining buffer.* Spin at 900 g for 5 min at 4 °C. At the second wash, spin down tube 1 together.
9. Resuspend nuclei from 3 tubes with 200 μl of *collection buffer*.
10. Pass nuclei through BD FACS tubes with 35 μm strainer top.

**Sorting**

Use 100 μm nozzle. Bulk sorting to 1.5 ml Eppendorf tubes preloaded with 200 μl of *collection buffer*.

Sorting with the following parameters:

1. FCS/SSC

2. 405-450/50 A/W, linear

3. SSC versus 561-575/26-A.

*RNase inhibitor. We use the one from Clontech (cat. No. 2313A). Although the RNase inhibitors from other brands might be interchangeable.

**We routinely use ~ 30 mg of pancreatic tissue.
