# Supplementary material for: NKX2-2 based nuclei sorting on frozen human archival pancreas enables the enrichment of islet endocrine populations for single-nucleus RNA sequencing
Source: BMC Genomics. 2024 Apr 30;25:427. doi: 10.1186/s12864-024-10335-w (PMC11059690; doi:10.1186/s12864-024-10335-w)
Supplement: Supplementary file 3 — Supplementary Material 3. [file 12864_2024_10335_MOESM3_ESM.docx]

Supplementary Table 1. Donor information.

| **Donor ID** | **RRID** | **Sex** | **BMI** | **Age (years)** | **Race** | **HbA1c** | **Cold Ischemia**  **Time (hours)** | **Sample Storage Duration (years)#** | **Experiment** |
| --- | --- | --- | --- | --- | --- | --- | --- | --- | --- |
| nPOD6407 | SAMN15879460 | Female | 16 | 4.6 | Caucasian | 5.5 | 13 | 4.6 | snRNA-seq with nuclei isolation by GRO-seq vs. citric acid |
| nPOD6292 | SAMN15879346 | Male | 19.3 | 3 | Caucasian | NA* | 20 | 15.3 | snRNA-seq with or without endocrine population enrichment |
| HPAP080 | SAMN19842609 | Male | 35.71 | 22 | African American | 5.4 | 19 | 1.3 | snRNA-seq vs. scRNA-seq |

*NA, not available.

# Sample Storage Duration is calculated as the years passed between sample collection and sample processing in the snRNA-seq experiment.
