# Supplementary figures and images for: NKX2-2 based nuclei sorting on frozen human archival pancreas enables the enrichment of islet endocrine populations for single-nucleus RNA sequencing
Source: BMC Genomics. 2024 Apr 30;25:427. doi: 10.1186/s12864-024-10335-w (PMC11059690; doi:10.1186/s12864-024-10335-w)

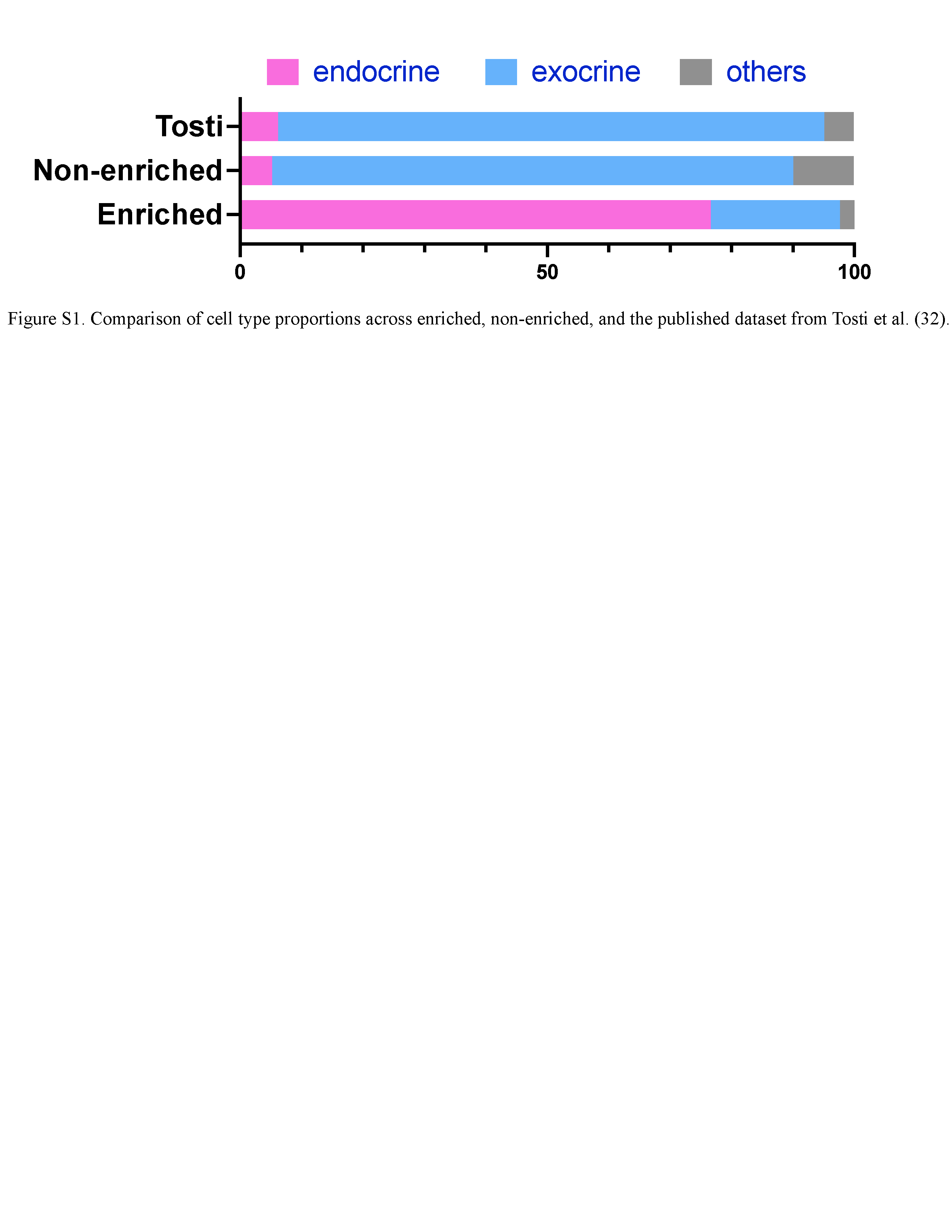

Supplement: Supplementary file 6 — Supplementary Material 6. [file 12864_2024_10335_MOESM6_ESM.tif]

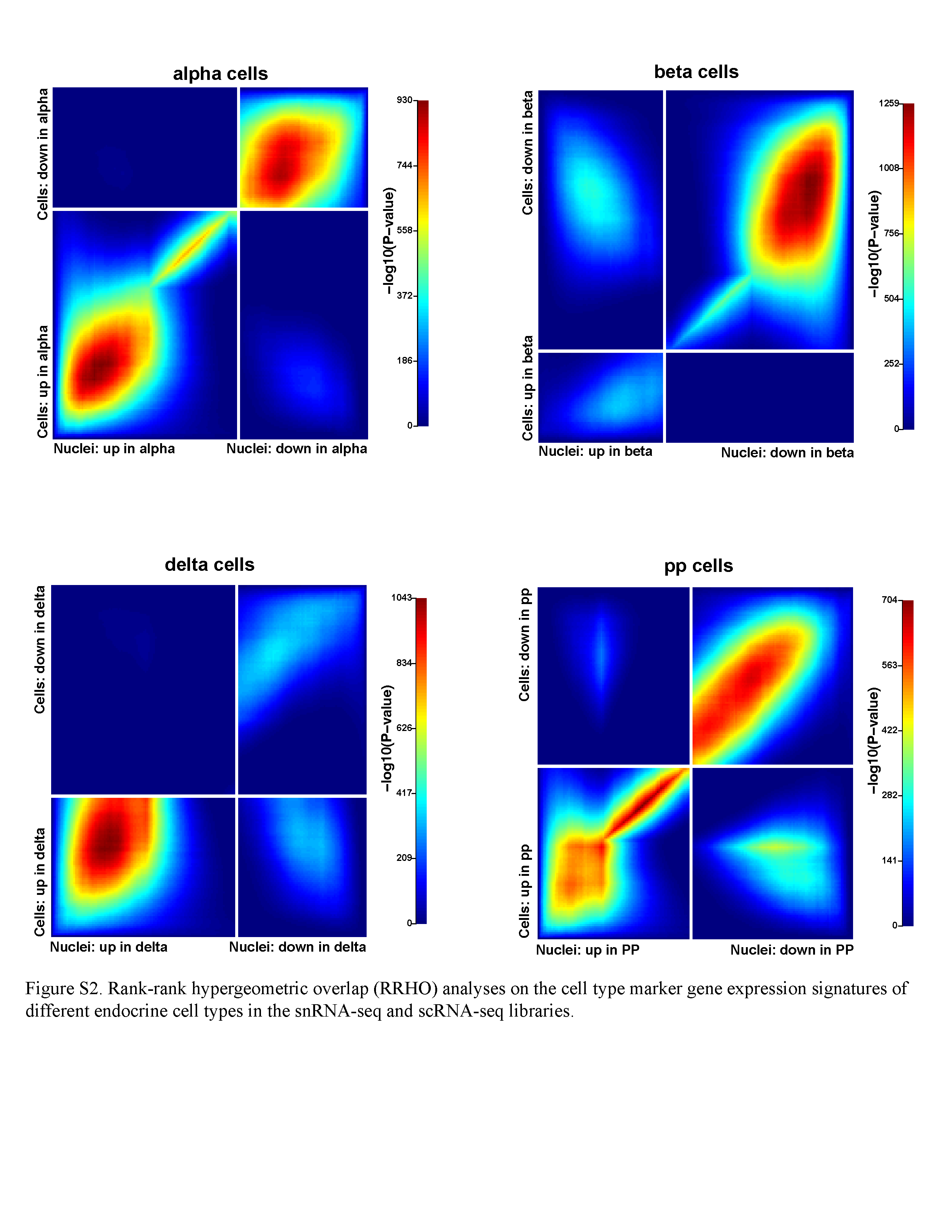

Supplement: Supplementary file 7 — Supplementary Material 7. [file 12864_2024_10335_MOESM7_ESM.tif]
